# Embodied skillful performance: where the action is

**DOI:** 10.1007/s11229-020-02986-5

**Published:** 2021-01-28

**Authors:** Inês Hipólito, Manuel Baltieri, Karl Friston, Maxwell J. D. Ramstead

**Affiliations:** 1 Berlin School of Mind and Brain and Institut Für Philosophie Humboldt, Universität zu Berlin, Berlin, Germany; 2 Wellcome Centre for Human Neuroimaging, University College London, London, UK; 3 Lab for Neural Computation and Adaptation RIKEN Center for Brain Science Wako, Saitama, Japan; 4 Mind, Brain Imaging and Neuroethics, Institute of Mental Health Research, University of Ottawa, Ottawa, Canada; 5 Division of Social and Transcultural Psychiatry, Department of Psychiatry, McGill University, Montreal, QC Canada; 6 Culture, Mind, and Brain Program, McGill University, Montreal, QC Canada

**Keywords:** Optimal control theory, Instructionism, Motor representation, Action-oriented representation, Active inference, Skillful performance

## Abstract

When someone masters a skill, their performance looks to us like second nature: it looks as if their actions are smoothly performed without explicit, knowledge-driven, online monitoring of their performance. Contemporary computational models in motor control theory, however, are *instructionist*: that is, they cast skillful performance as a knowledge-driven process. Optimal motor control theory (OMCT), as representative *par excellence* of such approaches, casts skillful performance as an instruction, instantiated in the brain, that needs to be executed—a motor command. This paper aims to show the limitations of such instructionist approaches to skillful performance. We specifically address the question of whether the assumption of control-theoretic models is warranted. The first section of this paper examines the instructionist assumption, according to which skillful performance consists of the execution of theoretical instructions harnessed in motor representations. The second and third sections characterize the implementation of motor representations as motor commands, with a special focus on formulations from OMCT. The final sections of this paper examine predictive coding and active inference—behavioral modeling frameworks that descend, but are distinct, from OMCT—and argue that the instructionist, control-theoretic assumptions are ill-motivated in light of new developments in active inference.

## Introduction

Expert performance dazzles us. The performance of a dance, of a musical piece, or of martial arts brings before us a display of human skills that, from a cognitive perspective, can only result from extensive practice. As opposed to bare movements, such as breathing and blinking, skillful performances are intelligent bodily activities, which harness knowledge about how to perform certain movements expertly.

This knowledge, however, is not always ready-to-hand in an explicit fashion, if at all; and indeed, explicit conscious monitoring of one’s performance while it is still ongoing often leads to ‘choking’. Choking under pressure occurs when failing to complete a task that has already been mastered. Although deliberative strategies sometimes lead to better performance, in real-world and do-or-die scenarios, thinking about the process or the outcome can lead to worse performance results^1^ (Cappuccio and Ilundáin-Agurruza 2020; Cappuccio et al. 2019a, b). Thus, we claim, what is involved in skillful action is not explicit knowledge.

Skillful performance—as a fine-grained bodily response to salient features of an ever-changing situation—can be described in terms of norms, knowledge, and expertise. This motivates a tendency to think of skillful action exclusively in terms of normative knowledge. However, it does not follow that bodily performance is itself the result of acting according to an explicit norm. Intelligent behavior, beyond deliberating and thinking, also involves intending, perceiving, understanding others, and so on. The remarkable, intelligent behavioral adaptation in the bodily performance of a skill suggests an understanding of knowledge beyond mere cognitive theorizing.

This brings the case of skillful performance to highlight for us a seemingly paradoxical relation to knowledge: *action both requires and is inhibited by it*. Skillful performance involves both an exquisite sensitivity to cultural norms and situational context. Thus, the impulse to think that knowledge somehow becomes internalized through practice without leaving the intellectual domain. However, as we observe in the phenomenon of choking, the explicit use of knowledge also seems to hamper expert performance. How then to make sense of the relation between knowledge and skillful performance? Surely knowledge is involved in skillful performance. But is it entirely in the form of a theory that ought to be executed as a top-down instruction?

Philosophers of cognition and cognitive scientists tend to model (skillful) action as being essentially of a theoretical nature, in the sense that it is mediated by knowledge. Skilful acting is, in this view, a matter of the brain ‘knowing’ (in a very strong sense) what actions need to be executed. That is, a cognitive top-down instruction, in the form of a belief, instructs the lower level motor system to behave in a certain way, giving rise to a skillful performance.

Following Wheeler and Clark (1999), we will refer to this position as *instructionism*. We cast instructionism in terms of *explicit instructions*, that is, *forms of knowledge that directly guide performance*. Applied to skillful performance, the instructionist assumption prescribes that instructions are harnessed in separable structures, such as beliefs, that are internal to the performing agent (Jeannerod 1997, 2006; Jankovic 2019; Stanley and Williamson 2017; Mylopoulos and Pacherie 2017, 2019; Pavese 2019; Piñeros Glasscock 2019). In more detail, the instructionist assumption says that skillful performance is enabled by *motor representations*, which harness knowledge about how a specific skillful performance *should be* executed in the form of *instructions* for movement. Instructionism, then, is the view that skillful performance depends on the capacity of an agent to *represent to itself* explicitly the procedure to be accomplished. In a nutshell, the instructionist assumption is representational in character—skillful action is driven by motor representation (Levy 2017; Schack and Frank 2020).

Indeed, the construct of motor representation has been cashed out in different, sometimes overlapping ways. In the philosophy and cognitive science literatures, we find flavors of this construct variously formulated either as propositional (Stanley and Williamson 2017), or as “practical representations” (Pavese 2019), “action-based ways of thinking” (Peacocke 1986), “ability-entailing concepts” (Stanley 2011), “executable concepts” (Pacherie 2011), “genic representation” (Wheeler and Clark 1999), “action-oriented representations” (Clark 1997; 2015b), and so on. What diverse accounts – as far apart as the propositional (Stanley and Williamson 2017) or the practical (Pavese 2019)—of motor representation have in common is that they are knowledge-driven. That is to say, they make of action as essentially a theoretical activity. Assuming motor knowledge is harnessed in internal structures that theorize explicit instructions for movement, and they will be our focus here.

To serve naturalistic purposes, in computational science, action is often studied under the rubric of *optimal control theory* (OCT) (Stengel 1994; Anderson and Moore 1990). OCT is a field in mathematical optimization that deals with finding a control for a dynamical system. Optimal motor control theory (OMCT) is a label for the modeling tools used to study motor behaviour and its neural processing. We target specifically OMCT to show that it rests on the instructionist assumption that the brain literally contains and leverages explicit instructions for movement. These models instantiate modularity (Fodor 1983)^2^ and the *separation principle* (Baltieri and Buckley 2018), since they take motor control to be realized by concerted processes performed by separable, modular subsystems (we will return to this below, in Sect. 6.1.) According to (linearly separable) OCT, skillful performance—indeed, all motor control—is realized computationally by three separate modules: (1) the inverse model, (2) forward model, and (3) state estimator (Friston 2011). OCT is instructionist in that it posits that skillful performance is realized through the construction and execution of an explicit *motor command*, which harnesses knowledge about (instructions for) skillful, knowledge-driven motor task execution. Thus, on this model of motor control, the so-called forward model and optimal controller work together to select an optimal action, based on a value function specified in terms of desired states; where the motor command is specified in terms of instructions for movement formulated in an intrinsic frame of reference (i.e., formulated in terms of the states of motor effectors, such as stretching and compressing of muscle fibbers).

The aim of this paper is to discuss critically the limitations of instructionist control-theoretic models of skillful performance. More specifically, we target the plausibility of separable, modular forward and inverse models and estimators responsible for the selection of actions based on a (value) function of future states, as postulated by OCT. The first section of the paper characterizes the instructionist assumption, which casts skillful performance as being based in the construction and execution of explicit motor representations. The following two sections characterize the implementation of motor representations as motor commands (OMCT). We attempt to show that its instructionist assumption is ill-motivated. The brain does not literally contain a detailed list of instructions that it uses to move the body. The final section of this paper leverages work in the active inference and sensorimotor frameworks—behavioral modeling frameworks distinct from OCT—to understand how skillful performance unfolds based on embodied interaction with environment. The account portrays skill as enacted without the need to assume it is driven by instructions couched in terms of the content of internal representations. The alternative interactionist account proposed here achieves this by avoiding two sets of commitments. It does not assume that generative models used in the active inference framework are models that are used by organisms or systems themselves. It does not assume that the explanatory story offered by sensorimotor accounts need posit any causally efficacious mediating knowledge.

## The instructionist model of skillful performance

In this section, we examine the commitments of instructionism. Instructionist models define motor control of the kind involved in skillful performance as the execution of a set of *instructions* for movements to be executed according to a prespecified method or procedure. A *motor representation* is defined as a structure internal to an agent that encodes, lists or otherwise harnesses a set of explicit instructions for movement, the execution of which leads to skillful performance. As we will see, such a motor representation prescribes the specific manner in which a task is to be accomplished.

How should we make sense to this? What does it mean for a thing to explicitly represent some state of affairs? It is common in the philosophy of mind to argue that representations involve modes of presentation (Frege 1892; Millikan 1997). This construct of mode of presentation has two main components: a representation presents some state of affairs (1) as being a certain way (2) from a specific vantage point. For instance, when I visually perceive the presence of a red apple, I perceive it from a certain *point of view* (i.e., from my visual vantage point), precisely *as being a red apple* (i.e., as opposed to perceiving it as being, say, a fruit or as being a red object). To represent a state of affairs thus entails that we represent it in a perspectival way as being a certain way; which is equivalent to saying that representations, essentially, must have a mode of presentation (Millikan 1991; Zalta 2001; May 2006; Burge 2010), i.e. a “Fregean presentationalism” (Sacchi 2018). In a nutshell, if there exist motor or practical representations, there must also exist a motor or practical mode of presentation (Glick 2015).

The modes of presentation at play in perception, thought, and action involve a set of (perceptual, conceptual, and motor or practical) *abilities* that constitute a *motor or practical perspective* (Pavese 2019; Burge 2009, 2010; Prosser 2019). Pavese’s (2019) discussion of representations situates what she calls practical representations (which we equate to motor representations as defined above) with respect to other kinds—perceptual and conceptual representations. The different varieties of representation differ in the manner in which they enable agents to represent states of affairs. Consider, e.g., the nature of perspectives that are involved in the perceptual representation of a situation. On this account, perceptual abilities (e.g., being able to discriminate between a middle C and a D sharp) constitute a perspective from which one can perceive states of affairs in the world; in this case, a musical state of affairs about the key of a song. To be endowed with such perceptual abilities enables an agent to *track states of affairs* in the world from a given perceptual perspective opened by these abilities (Dretske 1988; Millikan 1984; Fodor 1983). Conceptual representations, similarly, are related to the conceptual abilities with which agents represent states of affairs to themselves conceptually (Margolis and Laurence 1999; Machery 2009; Peacocke 1992; Prinz 2004). To represent some state of affairs conceptually thus entails the existence of a conceptual perspective, itself rooted in the conceptual abilities of the agent.

Importantly, this account allows us to fix the *content* of a representation, namely, the state of affairs that the representation is *about*, i.e. that which is disclosed by the relevant set of (perceptual and conceptual) abilities with which an agent is endowed—and thereby constituting the perspective from which it can represent that content. In the perceptual and conceptual cases, what is represented is the state of affairs that can be represented as being a certain way thanks to the perspective that is opened by the perceptual and conceptual abilities with which an agent is endowed; i.e., the state of affairs that is perceived or that is entertained in thought or predicated, respectively.

Pavese (2019) extends this line of reasoning to practical representation. Similarly, to perceptual and conceptual varieties, practical representations also represent by virtue of a set of *motor or practical abilities* that constitute a perspective from which state of affairs in the world is represented practically, in a format amenable to motor control. Practical abilities are defined as abilities to execute an action in a prespecified and typified manner. The content of a practical representation is a *method*: a specific sequence of physical movements to be carried out by the agent (Wolpert 1997; Pavese 2019, 2015). To be more precise, a method decomposes a particular task to be executed into component actions, perhaps nested the ones within the others, that when orchestrated bring about the desired outcome (Pavese 2019, 2015; Mylopoulos and Pacherie 2017). Thus, to explicitly represent the world from the perspective provided by practical abilities means to represent a task as having to be accomplished practically in a prespecified manner, i.e., according to the method or procedure by which the content of the representation—the task—is presented. The distinctive feature of practical representation is their ‘direction of fit’: they function to make the state of affairs in the world fit with the prescriptions harnessed in the practical representation (Pavese 2019). Whereas perceptual and conceptual abilities have a world-to-mind direction of fit, practical representations have a mind-to-world fit, which is what gives such representations their practical aspect.

Mylopoulos and Pacherie (2017) provide a definition of *motor representations* that dovetails nicely with Pavese’s (2019) account of practical representations and computational neuroscience research in motor control (Jeannerod 1997, 2006). In sum, they argue: (1) that motor representations represent objects and situations in terms of their *properties relevant for action*, in a *proprietary format* specified in terms of an *intrinsic frame of reference*—defined, e.g., by the state of motor effectors, muscle fibber extension and contraction, etc.; (2) that these motor representations are informed by or contain implicitly some knowledge about the body’s *biomechanical and kinematic constraints*; (3) and that motor representations—at least usually—serve the execution of *transitive movements*, specified in terms of an *extrinsic* frame of reference (i.e., a representation of states of affairs that is ‘objective’ in three-dimensional space rather than body-dependent).

The broad strokes of this definition seem common to most specific accounts of motor representation. For instance, on Pavese’s (2019) account, motor commands (which, as we will see below, implement motor or practical representations in OCT) represent the procedure or method according to which a task is to be accomplished, and are informed by a sensorimotor mapping from the actions being generated to their sensory consequences, satisfying condition (2). Moreover, they represent the method of task execution in a format that can both be used by the motor system to generate a motor action—i.e., in an intrinsic frame of reference, satisfying condition (1) —and also in a format that is sensitive to online, real time sensory feedback—i.e., in a manner that renders it responsive to outcomes specified in an extrinsic frame of reference, satisfying condition (3) of the definition just discussed.

Pacherie (2018) notes that motor representations meet criteria for representationality as set out by Bermúdez (1998): they have correctness or satisfaction conditions; they have a structure that exhibits and leverages some form of compositionality (i.e., evinces identifiable constituent or elementary units); and they also have a “grammar” that regulates the assembly of the constituent units into a coherent pattern. In cognitive science, this has led to the investigation of principles common to all skills, premised on the idea that what is thus common must be some set of representational processes. This view is labelled *intellectualism* (Stanley and Williamson 2017) and can be seen as the broader rubric under which falls our target in this article, namely, *instructionism*. At the root of such unifying models of skill is the instructionist assumption, which would allow for the construction of a general theory of skill, with epistemic attributes such as generativity, abstract rules or norms, and patterns of learning (Christensen 2019; Christensen and Sutton 2018).

Finally, we distinguish two kinds of instructionism (Wheeler and Clark 1999; Wheeler 2005), one strong and one weak. *Strong* instructionism is the claim that neural representations (in this case, motor representations) completely specify, on their own, the specific movements to be executed by an agent. We will see that this assumption is prevalent in many versions of motor control theory (e.g., Jeannerod 1997, 2006). The *weak* version of instructionism is the more modest claim that, among the many dynamically coupled systems that generate skillful performance (e.g., an able body, a normal ecological backdrop of cultural practices and standards, and so on) one kind stands out: structures internal to an agent that are responsible for encoding information that can be interpreted as explicit instructions for action, given a background of ecologically normal processes that enable them to play this role (Clark 1997; Engel et al. 2013).

On this more modest account, motor representations would play in the generation of behavior a role analogous to that of genes in the generation of phenotypic traits (Wheeler and Clark 1999). Genes however do not code for proteins (e.g., epigenetic transcription factors, the overall healthy and normal functioning of the cell, that cell’s being embedded in an organism, etc.) (Rosch et al. 1991; Godfrey-Smith 2007; Woodward; 2010; Griffiths and Stotz 2013; Hipólito and Martins 2017). Analogously, the weak instructionist framework for motor representation says that skillful performance is the result of an orchestrated process spanning components in the brain, body, and world, but that of these components, some special structures in the brain play the specific, explanatorily irreducible role of encoding explicit instructions for motor performance. Note, *en passant*, the conformity of this definition of representation with the definition of motor representation by Mylopoulos and Pacherie (2017) that was discussed above. In what follows, we will argue that neither kind of instructionism is warranted.

## From motor representations to motor commands

An appropriate scientific representational theory of motor action must elucidate both the kind of content in which motor representations traffic and, crucially, how such content is supposed to causally guide the generation of skillful performance—lest the story have no explanatory bite. Mylopoulos and Pacherie (2017) note that a scientifically respectable theory of motor action “cannot provide a full account of purposive action without appealing to motor representations and without explaining how intentions interface with motor representations.” (2017, p. 334). Computational models of OMCT must explain the manner in which motor representations are able to play the role of interface between the conative states of an agent (that is, desires and intentions to perform some task) and the motor performance.

Pavese (2019) argues that the construct of a *motor command*, which is widely used in the study of motor control, implements the construct of practical (or motor) representations in computational models of motor control. On this model, motor tasks are realized through a process involving “a series of sensorimotor transformations that map the intentions of the agent together with visual and other sensory information about the location of the targeted objects […] and the location of the limbs into a series of motor commands” (Pavese 2019, p. 791). On this view, a motor command is a practical or motor representation that enables the transformation from conative states or intentions of a motor agent (i.e., the agent’s intention to perform a task according to a prespecified method) to the actual motor performance itself (i.e., to the sequence of muscle movements that together comprise the skillful action).

On Pavese’s (2019) denotational model, the *content* of a motor command is the task to be performed itself; a view which finds echoes in related theories of motor representation (e.g., Wolpert 1997). More precisely, the content of a motor command is the task outcome. hat the task is meant to accomplish; e.g., moving one’s body to some location in space. The motor command thus comprises the specification of the outcome of a task in an *external frame of reference* (i.e., in terms of movement in three-dimensional space). A motor command is thus the *output* of a (conative) system responsible for motor planning.

Thus far, we have discussed what the contents of motor or practical representations are: they represent a specific *method* or *procedure*, which is defined as the explicit specification of movements in three-dimensional space (i.e., limb movements prespecified by a method or procedure, and harnessed as instructions for movement in an intrinsic frame of reference) that lead to some desired task outcome. We also examined how such practical representations get their content through their coupling to those practical abilities that open up a practical or motor perspective. The *mode of presentation* of a motor command is the *prespecified method* according to which the task is to be carried out. Thus, motor commands are also the *inputs* of the system that controls motor actions (Fridland 2020). They stand as an intermediary between the cognitive system of the motor agent (intention and desire) and the motor system responsible for carrying out the actual motor performance that ends up being executed.

Crucial to note is that, in order to play the intermediary role of informing the motor plant about what movements it must execute, motor commands must be generated via the inversion of a process mapping consequences in an extrinsic frame of reference, in which the desired movement is specified in terms of a task outcome in external coordinates (e.g., moving my finger to a point in three-dimensional space), from an intrinsic frame of reference, specified in terms of muscle movements. This entails an *inverse inference problem*, which requires working back from the desired sensory consequences (e.g., desired visual and proprioceptive sensory feedback) to a specification of their motor cause in an intrinsic frame of reference (i.e., a set of muscle activations that can generate such desired consequences). In other words, given some goal state that is specified in terms of extrinsic coordinates (and given conative states like desires and intentions), the problem to solve is the generation of a sequence of muscle movements, explicitly specified intrinsically in terms of stretching and compressing of muscle fibers. This has been called the “interface challenge” (Butterfill and Sinigaglia 2014). In other words, how are motor representations implemented such that they can realize or cohere with the intentions of an agent while also instructing motor performance?

## Motor commands and their representational role in optimal motor control theory

In this section, we examine how motor representations are implemented as motor commands in computational models of motor control under optimal control theory (OCT). Optimal motor control theory (OMCT) is a label we use to refer to the modelling tools of motor behaviour and its neural processing. We will see that the instructionist assumption that motor behaviour is underwritten by the construction and execution of explicit motor representations that are implemented in the brain as motor commands is, as it turns out, a pervasive one in studies of motor behaviour.

**Fig. 1:**
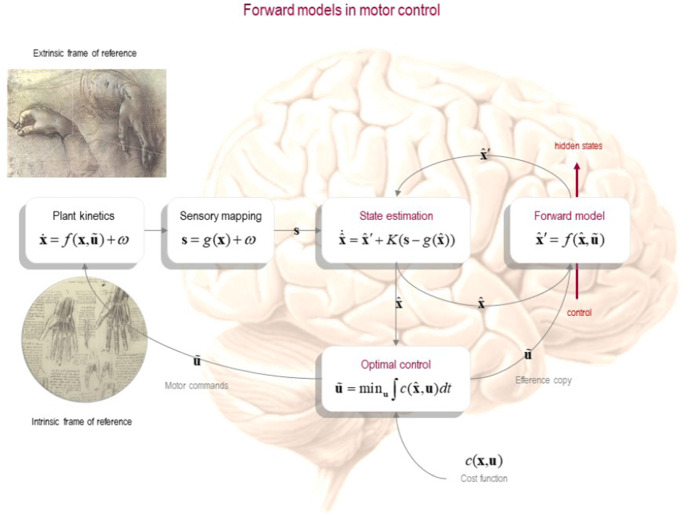
A computational model of optimal control. This figure presents a schematic of the computational architecture that underwrites optimal control theory. Note the separate optimal control or inverse model, state estimator, and forward model and the use of a cost function by the optimal control. Reproduced from Friston (2011)

This inverse inference discussed in the previous section—to wit, the problem of inferring how to specify muscle movements in an intrinsic frame of reference that bring about a goal state specified in an extrinsic frame of reference—is a nontrivial one, which has been addressed and finessed by OMCT. A general schema as how motor control is implemented in OMCT is depicted in Fig. 1.

**Fig. 2:**
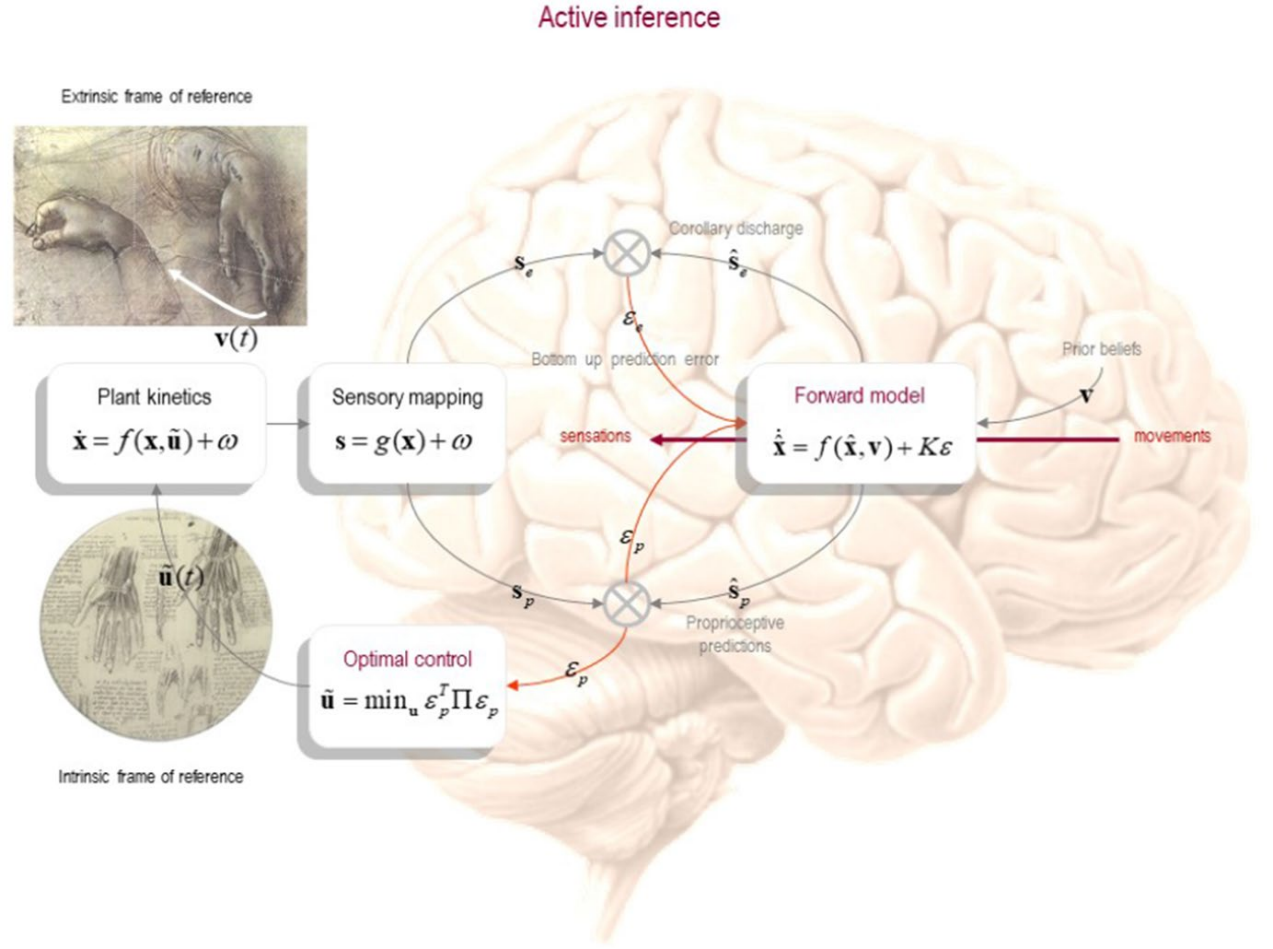
Motor control in active inference. This figure presents the models employed in the active inference framework. Note that the cost function has been replaced with proprioceptive prediction-error based control and that the separate inverse-forward models and state estimator have been merged into an expanded forward (generative) model. Reproduced from Friston (2011)

In OMCT (Wolpert 1997; Kawato 1999; Todorov 2004; Scott 2004; McNamee and Wolpert 2019), there are four main components at play in the generation of motor action: the motor plant, the state estimator, the forward model, and the optimal control (also called the inverse model). The motor control scheme functions, heuristically, as follows. The core of the model is the *optimal controller*, which tackles the inverse problem that was just discussed (hence, its other name, the inverse model). The optimal controller maps desired trajectories, specified in extrinsic coordinates, to muscle movements (i.e., to changes in muscular states specified in terms of intrinsic coordinates). The optimal controller selects an action based on the minimization of a *cost function*: the action that is selected is the one that leads to outcomes associated with the lowest cost or, equivalently, that leads to the most valuable states. The output of the controller is a *motor command*, which in our reading is a kind of practical representation, as discussed above.

Once an action is selected by the optimal controller—i.e., once the controller has constructed a motor command—the latter is sent to the *motor plant* for execution. The motor plant is the physical motor system (e.g., a limb) that executes the task to be performed; it carries out the movement prescribed by the motor command, which contains a specification of the muscle movements needed to realize the task outcome (a representation of the method, in the parlance of practical representation theory). Thus, the optimal control generates motor commands, which implements a specific method or procedure as specified in terms muscle movements in an intrinsic frame of reference (the motor command). It follows that the motor command qualifies as a motor or practical representation in the sense discussed above.

Physical movements of the motor plant, in turn, generate sensory information. This information is conveyed to a *state estimator*, via a sensory mapping. The function of the state estimator is to infer in what state the system finds itself, given its sensory feedback. The state estimator, technically speaking, comprises a probabilistic mapping from hidden parameters and states (i.e., hidden causes) to sensory observations; and its inference process inverts this mapping, to infer the most probable hidden cause, given available sensory data.

As the motor command is being relayed to the motor plant, a copy of the motor command, known as an efference copy, is sent to a *forward model*. Actions have sensory (e.g., visual and proprioceptive) consequences; and accordingly, the function of the forward model is to improve the execution of action by helping to finesse the inferences of the state estimator. Forward models do this by converting the (efference) copy of the motor command generated by the optimal control into a prediction of its sensory consequences, which can be discounted in state estimation. In effect, the state estimator uses information, pooled from the motor plant (via the sensory mapping) and the forward model, to form a prediction error: it compares the sensory outcome predicted by the forward model with the actual sensory data that is receives from the motor plant. It uses this error to finesse its posterior state estimates. Of note is that, in optimal motor control schemes, this prediction error is not typically represented in the model explicitly with a distinct variable or parameter; in Fig. 1, it is denoted as the update term **s**—*g*(**x**) weighted by the (Kalman) gain *K*. Finally, posterior state estimates are used to guide the process of action selection that is carried out by the optimal control; which brings us to where we began.

The standard approach to computational models of separable subsystems is based on linear quadratic gaussian (LQG) control (Stengel 1994). LQG-based models focus especially on formulations of perception and action in terms of (Bayesian) inference on the hidden states of the environment and on (deterministic) optimal control of a motor system (i.e., the body). Following this architecture, perception is often implemented using Kalman filters or similar Bayesian methods for estimation; while action is modelled as a process of feedback control based on linear quadratic regulators. The applications of the LQG framework in optimal motor control are ubiquitous, but often only implicit, with a few major exceptions more directly advocating its use in cognitive (neuro)science (Todorov and Jordan 2002; Todorov 2004; McNamee and Wolpert 2019).

## The instructionist assumptions of optimal control theory

The formulation of sensorimotor control in terms of OMCT heavily hinges on two different, but highly interconnected, assumptions: (1) the central specification of descending motor commands, and their (efferent) copies, in the form of detailed low-level instructions for control of the motor plant, which is specified in terms of an intrinsic frame of reference (i.e., extension and contraction of muscle fibbers), and (2) a separation of forward and inverse models, operating on complementary aspects of action planning and execution.

As highlighted in the previous section, the constructs of motor commands and their efference copies are typically used in frameworks focusing on the computational role of various components (the state estimator, forward and inverse models) derived from (optimal) control theoretic approaches to the problem of motor control. In this light, motor commands are cast as the product of an optimal controller (or inverse model), which builds accurate action policies based on explicit internal models of the biomechanical and kinematic properties of an agent’s musculoskeletal system (the sensory mapping). While forward models are thought to emulate the mechanical properties of a body and its interactions with an environment, once a certain action policy is implemented, inverse models are normally portrayed as inverting these cause-effect relationships to form plans over future actions, based on state estimators (also called comparator models) that combine internal simulations of agent-environment couplings and desired target states.

Let us recall the main features of the construction of motor command as it figures in OMCT. The presence of these two models, forward and inverse, naturally introduces the idea of different frames of reference over which internal models must operate: an intrinsic one, specified in terms of musculoskeletal properties of the body (e.g., muscle fibbers), and an extrinsic, movement-based one, characterising the external features of motor programs (e.g., hand position); see Friston (2011) for a discussion of these ideas in the literature. In particular, a forward model takes a system from an intrinsic to an extrinsic frame, predicting the effects of different movements using musculoskeletal plans specified by neural activity, and essentially translating motor commands into actions on the world and their consequences. On the other hand, an inverse model builds motor commands by inverting this causal chain. The inverse model first leverages a value function of states, to form a mapping from desired target states in an extrinsic frame of reference (i.e., in a coordinate system based on external consequences of movements) to a set of intrinsic coordinates in the space of muscle fiber activations; and then maps these activations to a set of neural activation patterns in the motor system that are capable of generating the appropriate and desired muscle activations. From a more mechanistic perspective, frameworks based on OMCT are sometimes characterized in terms of “force control,” stressing the idea that, in these models, motor commands specify actions in the form of muscle forces and joint torques (Hollerbach 1982; Kawato 1999; Ostry and Feldman 2003).

This architecture rests on the assumption, central to OMCT, that *value* (valuable states) is what *causes* action. As we have discussed, in models from OMCT, sequences of actions are selected according to a value function of states. This means that actions are selected by the optimal control that maximize the value of—or, equivalently, minimize the cost or risk associated with—future outcomes, defined in terms of desirable states.

A second major assumption in computational models of optimal control for action (OMCT) is their (often implicit) reliance on a sequential, modular architecture of perception-cognition-action, notably described as the “sense-model-plan-act” paradigm (Brooks 1991) or the “classical sandwich” of cognition (Hurley 2001); see Baltieri and Buckley (2018) for discussion. On this conception, action, perception, and cognition are depicted as separate processes, working relatively independently with specialized kinds of representations (practical, perceptual or conceptual, respectively) based on different mechanistic and neurophysiological (e.g., localised) implementations [cf. the idea of “vertical modularity” in (Hurley 2001)]. This is a classical idealisation of the sensorimotor loop, in which perception is portrayed as a bottom-up or feed-forward process with the primary goal of receiving information through the senses in order to build internal representations of the surrounding environment (Marr 1982). Action is then cast as a process of deriving appropriate motor commands based on the outcomes of cognitive internal manipulations, such as thinking and planning.

This notion of separable subsystems has its roots in the classical hypothesis of the modularity of the mind (Fodor 1983) and often constitutes one of the underlying assumptions in various applications of OCT to the study of cognitive agents (Wolpert 1997; Wolpert and Kawato 1998); see Baltieri and Buckley (2018, 2019a) and George and Sunny (2019) for some reviews. On the modularist view, more ‘peripheral' components of cognitive systems, i.e., those subserving action and perception (but according to some, perhaps also some of “central processing”, see Barrett and Kurzban 2006 for a review) are implemented as separable modules, working independently to transform sensations incoming through input interfaces (perception) into internal models, used to plan actions executed via output layers (motor control, behavior). The information content of each specialized module is encapsulated (i.e., the module is informationally semi-independent from other parts of a system), and the kinds of computations it performs is specialised as well; an idea closely related to the concept of cognitive impenetrability typically discussed in the context of perceptual processes (Pylyshyn 1999; Coltheart 1999; Barrett and Kurzban 2006; Raftopoulos 2019).

In summary, motor control schemes in OMCT are *instructionist*, as we described the notion in the opening sections. This can be seen from the modular architecture in these schemes, which is based on sensorimotor representations in the form of separable forward-inverse models and estimators. This architecture for motor control is used to compute explicit motor commands, which implement the construct of motor representation: they harness explicit motor instructions, canvassed in a proprietary format that the motor plant can use to guide an instruct the execution of action (i.e., specified in an intrinsic frame of reference), so obtain desired states specified in extrinsic coordinates. We now critically examine this assumption.

## Less control, more action: from optimal control to predictive coding and active inference

Thus far we have claimed that the instructionist assumption of OMCT has two parts:

1. The presence of *separate forward and inverse models*, with the latter being in charge of selecting motor plans according to a cost function expressing the value of states to be attained through action; and
2. Instructions expressed in the form of *motor commands,* lists of low-level motor outputs that are built using internal representations of the biomechanics properties of a motor plant, i.e., the body.

In what follows, we critically examine these two assumptions.

### From forward-inverse models and cost functions to generative models

The optimal control approach has been repeatedly challenged over the years, with work questioning its neurophysiological plausibility (Ostry and Feldman 2003; Feldman 2009, 2015; Latash et al. 2010; Latash 2020), the computational scheme of forward and inverse models with separate roles (Adams et al. 2013; Clark 2015a; Pickering and Clark 2014), its reliance on cost functions, and its claims regarding optimality expressed in terms of the value of states (Friston 2011; Friston et al. 2012a, b; Pezzulo et al. 2015).

The account of separable, modular perceptual and motor subsystems, in particular, has recently been suggested to reflect a classical result in the control theory literature, where modular regulators are defined using the “separation principle” (Baltieri and Buckley 2018, 2019a). In control theory, this principle describes a set of necessary and sufficient conditions for the independent optimisation of the two main components of a device regulating a system in the presence of uncertainty: a paired state estimator and forward model, and a (deterministic) controller. Under the assumptions of the separation principle, teleological behaviour can be cast as a sequential process of *optimal* estimation, combining state estimation and forward models, followed by a phase where internal world (forward and inverse) models are refined and used for off-line planning. This leads to an *optimal* control stage, where actions are produced by an inverse model of the dynamics of a plant (e.g., the body) using accurate estimates of the current state of a system. An intrinsic assumption of optimal motor control approaches based on the separation principle is thus that sensorimotor control is orchestrated mainly by two separate modules: a combined state estimator/forward model and an inverse model. The assumptions behind the separation principle in control are, however, rather strict and include, for instance, the presence of linear dynamics, the use of quadratic cost functions and dynamics where uncertainty is expressed using Gaussian noise. As previously suggested, some of these assumptions can be easily violated when applied to the study of biological systems (Todorov 2005; Baltieri and Buckley 2018).

Perhaps the most important shortcoming of this approach comes from the fact that its formulation expresses motor signals as neutral, or equivalently, the lack of *dual effects* of motor actions (Bar-Shalom and Tse 1974). In practice, this means that the canonical controls generated by LQG models cannot reduce (or even increase) a system’s uncertainty in the future, i.e., actions can only be instrumental, and have no epistemic effect on future state estimates—with a possible exception to this account found in the optimal feedback control extension of the model by Todorov and Jordan (2002) and Todorov (2005). In accordance with the differences in terms of epistemic actions, approaches based on the separation principle have variously been addressed also as adaptive (as opposed to dual) controllers (Kappen 2011a, b), or feedback (as opposed to closed-loop) methods (Bar-Shalom and Tse 1974).

The active inference framework^3^ offers an alternative account of skilled action (Friston et al. 2012a, b; Friston et al. 2017). In the active inference framework, some of the assumptions that underwrite the separation principle are dropped in favour of a more cohesive and unifying perspective on forward and inverse models [Baltieri and Buckley 2018, 2019a; see also George and Sunny (2019)]. The active inference framework thus comprehensively challenges the optimal control theoretic approach to sensorimotor behaviour, highlighting some of the limitations associated with such schemes based on value functions (Friston 2011; Friston et al. 2012a, b), with natural implications for accounts of skillful performance. In particular, here, we refer to the idea that traditional OMCT accounts of behaviour can only specify performance using a single number, a scalar that is defined and consequently tracked by a *value* function. Value functions express criteria of optimality for motor behaviour that instantiate an index of “accuracy,” which reflects how a specific definition of value uniquely maps to an act or motor plan conforming to a goal. Indeed, the physics of flow embedded in active inference accounts of sensorimotor behaviour show that motion in a biologically realistic state space irreducibly includes two orthogonal kinds of motion: an irrotational (or curl-free) component and a solenoidal (or divergence-free) component. The former is what allows the flow to climb a gradient towards more valuable or probable states (i.e., moving from less to more valuable states); while the latter specifies a flow around an isoprobability contour, where all entered states have an equal value or probability (i.e., different configurations of states expressing the same value, or rather skillfulness, that may be relevant in different contexts). Heuristically, the irrotational component contributes to the appetitive, motivated aspect to behaviour, getting the agent closer to desired states or observations; whereas the solenoidal component describes behaviour that does not aim directly at need satisfaction. Together, these flows provide a richer framework for expressing skilfulness as a process whose characteristics go beyond a simple gradient of value/accuracy. Value functions—and indeed any motor scheme based on functions that return scalars – are not up to the task of modelling the variety of (skillful) acts describing human behaviour because, by construction, they cannot account for the solenoidal aspect of flow.

The active inference framework does away with the possibility of positing inverse models, previously claimed to be physiologically not realisable (Ostry and Feldman 2003) and computationally intractable without extra constraints (Adams et al. 2013). The active inference framework replaces value functions and solutions to optimal control problems formulated as motor commands based on dynamic programming methods with *priors* (in the form of Bayesian ‘beliefs’). That is, the active inference framework replaces the inverse-forward model pair with a *generative model* that expands on a forward model to harness probabilistic beliefs about expected sensory consequences of action. Rather than using a separate inverse model to infer the most appropriate course of action, active inference schemes invert the generative model through the use of (approximate) Bayesian inference techniques in order to select action policies.

The active inference framework does not operate with value functions (Friston 2011; Friston et al. 2012a, b; Adams et al. 2013). Instead of selecting actions using a (value) function of states, active inference models directly construct a prior preference over sensory outcomes or observations, which is used to guide motor control in a feedback-sensitive, online fashion, in an extrinsic frame of reference. Technically, active inference extends popular predictive coding models used in neuroscience, where perception is cast in terms of prediction error minimisation (Rao and Ballard 1999). The active inference framework extends this account to model motor control, and explains action selection by appealing to the minimisation of divergence between predicted sensory data and actual sensory data in, e.g., visual and proprioceptive modalities. The core idea, then, is that rather than select an explicit motor command, the organism ‘infers’ what it must be doing, under the assumption that what it does must minimize prediction error (see Friston 2011). Crucially, this brings perception and action together in the same functional profile and also explains some of the similarities between functions of sensory and motor cortices (Adams et al. 2013). While this move from a problem of control to one of inference in terms of active inference does not make the problem mathematically easier in and of itself (Friston 2011), it offers a different model of skilled action which also respects the neurophysiological evidence.

In this light, the active inference framework stands in stark contrast to optimal control accounts described earlier, where forward and inverse models are seen as distinct functional units with perception and action lying at the two opposite ends of a chain of sequential processing (cf. the classical sandwich of cognition). The active inference framework does away with inverse and forward models in favour one single, expanded generative model (Fig. 2).

### From motor commands to proprioceptive predictions

A second important move afforded by the active inference framework is the replacement of motor commands in the form of accurate motor plans in intrinsic (bodily) coordinates, considered to be unrealistic due the required specificity of a plan and the huge number of degrees of freedom of the neuromuscular system, with predictions about proprioception (Ostry and Feldman 2003; Adams et al. 2013). This implicitly solves some of the main issues with models relying on the inversion of the many-to-one mapping from a high-dimensional intrinsic frame of reference to a low-dimensional external, movement-based, coordinate system. In practice, this summarises the problem of motor redundancy [see Latash (2012)] where several combinations of different muscle activations can lead to the same final goal, think for instance of an arm reaching task and the virtually infinite number of possible arm trajectories that could satisfy a given final goal in the form of a target location.

In the active inference framework, action planning is described in terms of an inversion process of a generative model via the inclusion of a proprioceptive modality, and an ensuing minimisation of proprioceptive prediction errors. While this proposal provides an alternative, arguably more parsimonious, version to inverse models, it only apparently solves the most problematic aspect of these models: the inversion of the process generating musculoskeletal motor plans from patterns of neural activity. The hard part still consists of ultimately explaining action execution via the inverse mapping from an extrinsic to an intrinsic frame of reference, for which predictive coding models don’t provide a natural account (Friston 2011). To solve this problem, the active framework inference forgoes explicit movement specification in terms of a mapping from an extrinsic to an intrinsic frame of reference. It does so by dropping its reliance on the value function that, in the end, in OMCT, specifies motor commands in terms of musculoskeletal properties of a system.

The active inference framework proposes an account of perception–action cycles that is consistent with some ideas of the mechanical description of motor actions provided in threshold or referent control (previously also known as the “equilibrium-point hypothesis” or “virtual trajectories control”) (Feldman 2015). Similarly, to this framework, active inference suggests that, rather than encoding muscle forces or joint torques, descending motor signals act as thresholds that shift the activations of stretch reflex muscles in order to create movement as a “chain of reflexes” (Adams et al. 2013). Unlike referent control however, active inference framework commits to the idea that such thresholds can be interpreted directly in terms of responses to proprioceptive information of the target state, as opposed to thresholds “lambda” typical of referent models (Feldman 2015).

In the active inference framework, proprioceptors become perception–action units whose combined functions for perception and action are controlled by precision parameters (Adams et al. 2013). This has two deep ramifications for motor control. First, in active inference, classical motor command and efference copy constructs of OMCT become redundant; and second, control assumes a dual role in active inference schemes, reflective of the dual role of action itself in these schemes. The former point speaks to the idea that frameworks based on optimal control and the separation principle typically require (efference) copies of motor commands (forces and torques) to be passed from an inverse to a forward model, such that predictions generated by forward models can discount the effects of one’s own actions on one’s perception of the world. While in robotics and control theory, this is classically solved by the presence of an efference copy of motor signals sent to the estimator (Kawato 1999) that is known to the engineer/roboticist; in neurobiology, on the contrary, the role of this copy is hotly debated (Bridgeman 2007; Feldman 2009, 2016; Adams et al. 2013). Thus, for principled reasons, the active inference framework avoids the requirement for a controller to send an efference copy to the estimator and forward model. This is due to the fact that forward connections already denote prediction errors in their mappings from prior beliefs about expected limb trajectories to their (proprioceptive) sensory outcomes.

Further, by building a framework that takes advantage of simple, lower-level motor functions, which are increasingly recognised as being more than simplistic, pre-programmed reflexes (Bizzi et al. 2000; Buhrmann and Di Paolo 2014; Weiler et al. 2019), the active inference framework introduces an account of the dual effects of action at different levels. On a short spatio-temporal scale (action execution), one finds an implicit account expressed in terms of variational free energy (or prediction error) minimisation,^4^ constrained by the dual role of proprioception in predictive coding models with reflex arcs (Friston et al. 2010). On longer time scales (such as those involved in action planning), on the other hand, a more explicit account of this exploration/exploitation problem emerges with the minimisation of *expected* free energy on expected future outcomes given prior preferences, and the explicit presence of epistemic and instrumental terms within the definition of the expected free energy functional (Friston et al. 2017).

Having offered reasons to reject both conditions, (1) the presence of forward and inverse models, as well as (2) instructions as motor commands, we now see that active inference can offer a formal model to explain skilled action, from first principles, without supposing explicit motor instructions.

## Motor control as interactive sensorimotor engagement with the world

Let us take stock of what has been said so far. We started from the observation that the most popular models in the field of motor control studies make an *instructionist assumption*. In instructionist models, skillful performance is explained by appealing to the construction and execution of motor commands. That is to say, these models posit motor or practical representations, which harness knowledge about how a specific skillful performance is to be executed in the form of explicit motor instructions that is specified in terms of an intrinsic (muscle-based) frame of reference. We then reviewed new frameworks in the study of motor control—namely, active inference and predictive coding—which undermine some of the instructionist assumptions. We saw that, in these frameworks, nothing like an explicit motor command ever needs to be computed; which undermines even the weak version of instructionism (Wheeler and Clark 1999). Where does this leave us in terms of a positive proposal? What is skillful performance, if it does not consist of detailed lists of instructions for the execution of motor commands?

The active inference framework offers a formal model of motor control as a process of online, real-time motor adaptation to an environment. Such adaptation can be understood in terms of *attunement* between organisms and their environments (Bruineberg and Rietveld. 2014; Anderson 2017; Ramstead et al. 2019; Hipólito 2019; Hipólito et al. 2020). The tight and reciprocal reconnection between perception and action in the active inference framework resonates deeply with several key ideas developed within *embodied* and *enactive* approaches to cognition and agency (Di Paolo et al. 2017; Newen et al. 2018; Gallagher 2020; Ramstead et al. 2019). In particular, the inescapable codependence between action and perception in active inference coheres nicely with one brand of enactive-embodied cognition, namely, *sensorimotor approaches* to the study of cognition (O’Regan and Noë 2001; Engel et al. 2013, 2015; Di Paolo et al. 2017; Gallagher 2020).

One might wonder how the active inference framework could complement and be used to support enactive-embodied approaches, as a realistic proposal about the nature of cognition. After all, the active inference framework is neck deep in talk of ‘inferences’, ‘predictions’, ‘prediction errors’, ‘priors’, and even ‘beliefs’. Prima facie, such references seem to entail a serious commitment to an understanding of cognition as a form of world-modelling. Accordingly, this appears to commit proponents of the active inference framework to the assumption that, when cognizing, organisms or their subparts must be making use of generative models in order to act on the world intelligently, because having a generative model is thought to be what enables cognizers “to evaluate potential actions using (as the name suggests) some kind of inner surrogate of the external arena … [something that allows them] to ‘navigate into the future’” (Clark 2016, p. 254).

Yet even some of the most forthright defenders of the active inference framework have backed away from making such strongly realist claims about the causally efficacious character of generative models. Indeed, Friston (2013) has advanced the view that an “agent does not have a model of its world—it is a model” (Friston 2013, p. 213). Elsewhere, in line with a recognition of the purely formal character of generative models, Friston et al. (2012a, b) advise that, “We must here understand ‘model’ in the most inclusive sense, as combining interpretive dispositions, morphology, and neural architecture, and as implying a highly tuned ‘fit’ between the active, embodied organism and the embedded environment.” (p. 6).

Crucially, if we accept the generative models of the active inference frameworks in line with these proposals then we must embrace the idea that generative models are causal in the sense of an agent’s enactive attunement with the world, rather than a part of the causal efficacious machinery *used by* an agent. A simple reason to do so is that a generative model cannot be both identified with the system itself and, simultaneously, a model that the system itself uses to drive its behaviors.

If we understand the status of generative models in the active inference framework as purely formal, epistemic tools, then this framework is, we argue, compatible with a sensorimotor approach to understanding skilled action. Sensorimotor accounts assume that perception (O’Regan and Noë 2001) and perhaps higher order cognitive functions (Maye and Engel 2013) emerge as a process of interactive engagement with the world, based on an organism’s acquired responsiveness to sensorimotor contingencies, defined as a series of invariant correlations describing the relations between sensory and motor modalities (Noë 2004). Perception is thus only appropriately defined for agents actively interacting with their milieu, when the world is dynamically coupled to an agent (Di Paolo et al. 2017); rather than on the “classical sandwich” of cognition (Hurley 2001), which casts motor control in terms of sequential perception, planning, and action. On this account, perception and action are cast as the *mastery* of sensorimotor contingencies. Importantly, we argue that it is possible to retain the key interactionist idea from the sensorimotor approaches in play without buying into any of the more controversial claims about the representational character of sensorimotor contingencies and their nature as invariant correlations for perception and action for mediating knowledge (see Hutto 2005, and Hutto and Myin 2013 for critiques of the latter).

Importantly, as suggested by Di Paolo et al. (2017), the sensorimotor view reflects a spectrum of ideas, which includes simple open-loop sensorimotor correlations, closed-loops ones, regularities given a goal, and optimal sets of regularities according to a certain performance metric. These can be understood using the tools of dynamical models of cognition, capturing the brain-body-environment interactions in terms of dynamical systems as opposed to assuming the agent is involved in symbolic computation (Chiel and Beer 1997). These “anti-representational” ideas hark back to the explanatory strategies of ecological psychology (Gibson 1979) and need only speak the “lawful linkages between sensory and motor systems” advocated by Rosch et al. (1991) or the “subjective physics” of perception (Brette 2013). When they are situated in the context of biological systems and their biomechanical constraints, sensorimotor contingencies may also be seen in terms of “synergies,” capturing the attunement of different muscle groups to specific tasks engaged by an agent (Latash 2008). Thus, instead of constructing elaborate instructions harnessed in motor representations, motor control deploys smooth real-time adaptation to the salient aspects of a situation, leveraging the biomechanics of interacting physical bodies.

In active inference, a similar account emerges once we consider non-modular approaches to cognition, combining predictive approaches to perception, dynamic reflex arcs, and mechanisms for planning over expected future outcomes (Parr and Friston 2018). As previously suggested, for instance, by Brette (2013) and Di Paolo et al. (2017), the idea of sensorimotor contingencies is well captured by simple relationships between proprioceptive sensations and motor actions. We further argue that the predictive role of proprioception advocated in active inference extends causally linear accounts of motor control (such as the one by Brette 2013), which tend to focus only on the contingency between new actions and their consequences on proprioceptive sensations (i.e., new action → new proprioceptive state). Active inference in fact proposes a complementary view, where predictions of expected proprioceptive states are not just seen as passive reactions to new motor signals, but as also triggering adjustable, dynamic reflex arcs to generate new actions (new proprioceptive state → new action → new proprioceptive state → new action → …). The temporal depth of this model confers a more active, anticipatory role to proprioception, now seen in a causally circular model of sensorimotor control, in line with the enactive and embodied approach of Di Paolo et al. (2017); where action is informed by perceptual processes and perception is itself an active process of engaging with the world (Baltieri and Buckley 2017, 2019b).

## Conclusion

This paper critically discussed the limitations of instructionist approaches to skillful performance and also to assess what kind of knowledge (if any) is involved in motor control. The instructionist assumption is that according to which skillful performance is, at bottom, driven by motor representations that harness instructions about how to perform a given task. We examined the manner in which motor representations are operationalized as motor commands in OMCT. We asked whether the assumption of modular knowledge-driven motor control in OMCT, which is based on a modular architecture implementing separable state estimators, forward models, and inverse models, is warranted, and concluded that it is not. We argued that active inference does not need to posit the instructionist assumption. If the generative models proposed within the active inference framework are understood as purely formal tools, it is possible to develop an interactionist account of skilled performance, i.e. an account where, by drawing on the resources of dynamical tools, perception and action are deeply connected. Future research will elaborate on the theoretical and formal consequences of active inference as a non-instructionist framework.

**Table:** Box 1: Active Inference and the free energy principle

*Variational free energy:* a statistical measure used in problems of approximate Bayesian inference as an effective upper bound to surprisal, a (usually incomputable) quantity that represents the negative log-probability of an outcome, e.g., the sensory states for an organism. Under Gaussian assumptions, variational free energy reduces to a weighted sum of prediction errors
*Free energy principle:* A statement of some of the properties that all self-organizing systems that have necessarily by virtue of existing. The free energy principle says that self-organizing systems that have a Markov blanket and a phenotype will appear to engage in behavior that minimizes a variational free energy functional
*Active inference:* Under the free energy principle, systems can be interpreted as engaging in active inference in order to minimize their free energy. A system can be described to engage in active inference in the sense of performing belief updating and acting such as to fulfil prior preferences about observations. Describing a self-organizing system in terms of active inference means that the system acts upon its external milieu to maintain itself in its preferred states (cf. homoeostasis). Active inference provides a mechanism to derive the dynamics of sensory and active states such that they minimize a variational free energy functional. This allows us to describe an agent as engaging in actions that will get them closer to their preferred sensory states. Belief updates, in turn, contribute to the optimization of internal states, which tightens the (free energy) bound on surprisal, thus enabling action to avoid (statistically) “surprising” sensations; and corresponds to perception

## Footnotes

1 Dimensions of self-consciousness, such as sensorimotor, affective, narrative consciousness, have been identified as a general factor of choking under pressure. See Cappuccio et. al (2019a, b); see also Gray (2020).

2 See also Drayson (2018), Jeannerod (2018), Levy (2017), Mylopoulos and Pacherie (2017) and Fridland (2015, 2017).

3 We will occasionally appeal to technical terms that are in common usage in this field. Please see the glossary in Box 1 for relevant definitions.

4 See Box 1.
